# Inactivation of MSMEG_0412 gene drastically affects surface related properties of *Mycobacterium smegmatis*

**DOI:** 10.1186/s12866-016-0888-z

**Published:** 2016-11-08

**Authors:** Anna Zanfardino, Adriana Migliardi, Daniele D’Alonzo, Angela Lombardi, Mario Varcamonti, Angela Cordone

**Affiliations:** 1 Department of Biology, University of Naples “Federico II”, Via Cintia, 80126 Naples, Italy; 2 Department of Chemical Sciences, University of Naples “Federico II”, Via Cintia, 80126 Naples, Italy

**Keywords:** Mycobacterium smegmatis, msmeg_0412, GPLs, Lipolytic, Cell wall, Biofilm

## Abstract

**Background:**

The outermost layer of mycobacterial cell wall is rich in lipids and glycolipids, surface molecules which differ among species. *Mycobacterium smegmatis*, an attractive model for the study of both pathogenic and non-pathogenic mycobacteria, presents glycopeptidolipids (GPLs). All the genes necessary for the biosynthesis of such molecules are clustered in a single region of 65 kb and among them, the *msmeg_0412* gene has not been characterized yet. Here we report the isolation and subsequent analysis of a MSMEG_0412 null mutant strain.

**Results:**

The inactivation of the *msmeg_0412* gene had a drastic impact on bacterial surface properties which resulted in the lack of sliding motility, altered biofilm formation and enhanced drug susceptibility. The GPLs analysis showed that the observed mutant phenotype was due to GPLs deficiencies on the mycobacterial cell wall. In addition, we report that the expression of the gene is enhanced in the presence of lipidic substrates and that the encoded protein has a membrane localization.

**Conclusion:**

*msmeg_0412* plays a crucial role for GPLs production and translocation on *M. smegmatis* surface. Its deletion alters the surface properties and the antibiotic permeability of the mycobacterial cell barrier.

**Electronic supplementary material:**

The online version of this article (doi:10.1186/s12866-016-0888-z) contains supplementary material, which is available to authorized users.

## Background

Mycobacterial cell wall is a unique well organized barrier which confers low permeability to different chemical agents and common antibiotics [1]. The envelope is waxy, hydrophobic, and thicker compared to that of other bacteria and is organized in two compartments, the inner layer, covalently linked to the plasma membrane and formed by peptidoglycan, arabinogalactan and mycolic acid, and, the outer layer, which is essentially formed by glycolipids and proteins [2, 3]. Glycopeptidolipids (GPLs) are the major surface exposed molecules found in various mycobacterial species including non-pathogenic (e.g *M. smegmatis*) and non–tuberculous pathogenic mycobacteria (e.g. *M. avium, M. intracellulare, M. abscessus*) [4, 5]. GPLs have a common fatty acyl-tetrapeptide core which is glycosylated with 6-deoxy-l-talose and variable *O*-methyl-l-rhamnose residues [6–8]. This is known as non-serovar-specific GPL (nsGPLs) and represents the main product of *M. smegmatis* GPLs. In *M. avium* GPLs present a more complicated structure in which an additional rhamnose and oligosaccharide residues are added to 6-deoxy-l-talose, resulting in serovar-specific GPLs [7, 9, 10]. These molecules, which may represent more than 70 % of total surface lipids [11], are required for cell aggregation, sliding motility and biofilm formation [12–15]. Moreover several studies reported that slight modifications of the GPLs structure are able to affect the mycobacterial ability to stimulate the host immune response, suggesting a role as antigenic molecules [16–19].

The fast growing *M. smegmatis* is frequently used as a model organism to study the molecular and physiological mechanisms used by slow-growing pathogenic mycobacteria. The *M. smegmatis* GPLs biosynthetic locus has been previously characterized either experimentally or by *in silico* prediction [20–23]. Most genes of the *M. smegmatis* GPL locus are conserved in two clinically significant mycobacterial species such as *M. abscessus and M. avium* subsp. *paratuberculosis* [5, 24].

Among them, the gene *msmeg_0412* is still uncharacterized and its product shares 66 % and 67 % identity with the predicted protein MAB_0402, and MAP2244 from *M. abscessus and M. avium subsp. paratuberculosis*, respectively. Interestingly, *M. tuberculosis*, a mycobacterium which does not produce GPLs on the cell wall, presents Rv1184c which shares 45 % identity with MSMEG_0412. Rv1184c has been recently characterized as the Acyltransferase Chp2, essential for the final biosynthetic steps of polyacyltrehalose (PAT), a pentaacylated, trehalose-based glycolipids only found on the surface of pathogenic mycobacteria [25, 26].

In this study we show that the inactivation of *msmeg_0412* gene alters the lipid profile surface of *M. smegmatis* and enhances the bacterial susceptibility to antibiotics. In addition, we report that *msmeg_0412* gene expression is enhanced in the presence of lipidic substrates and that the encoded protein localizes on the cell wall. Taken together, our results indicate that MSMEG_0412 is an important player in the complex GPLs biosynthetic process in *M. smegmatis* and allow speculating that MSMEG_0412 is translocated to the cell wall where it contributes to GPLs synthesis.

## Results

### Deletion of the *msmeg_0412* gene alters *M. smegmatis* surface properties, enhances suscepitibility to antibiotics and affects GPLs synthesis

**Fig. 1:**
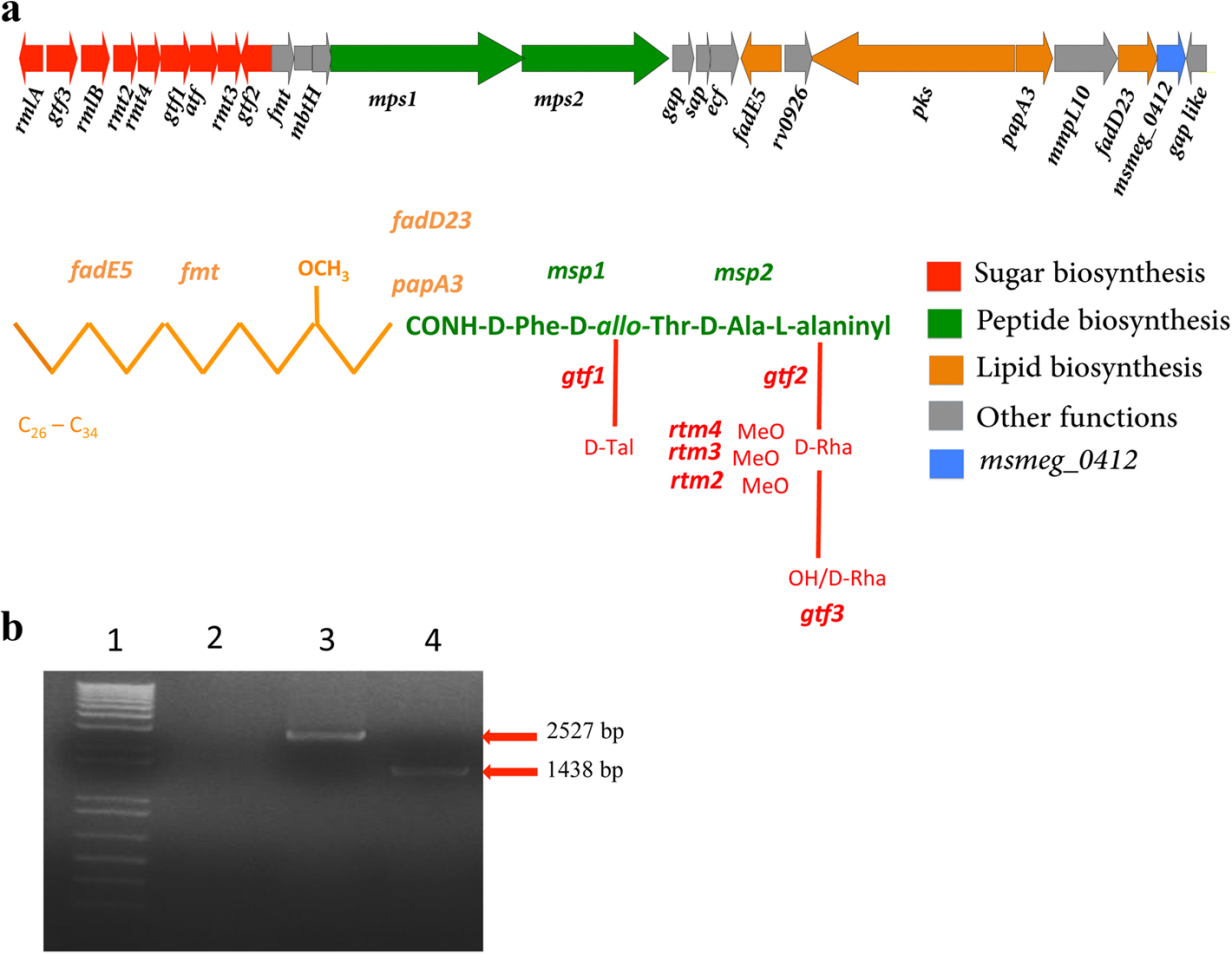
**a**) Schematic representation of the GPLs structure from *M. smegmatis* and its biosynthetic *locus* on the chromosome. The genes involved in the synthesis of lipid moiety (*orange*), peptide (*green*) and glycosylation (*red*) of GPLs are indicated. **b**) Agarose gel electrophoresis showing PCR-based validation of the *msmeg_0412* gene deletion in the GM1 strain. Lanes: 1) DNA Ladder marker (1Kb); 2) PCR negative control; 3) wt Chromosomal DNA used as template (2527 bp-amplicon expected using 0412-F1 and 0412-R2 as primers); 4) GM1 Chromosomal DNA used as template (1438 bp-amplicon expected using 0412-F1 and 0412-R2 as primers)

**Fig. 2:**
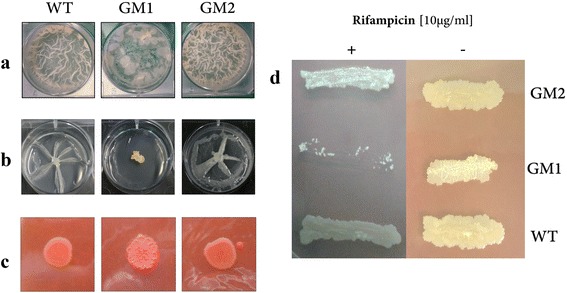
Phenotypic analysis of *M. smegmatis* strains. Wild-type strain was compared to *msmeg_0412* null mutant (GM1) and complemented (GM2) strains for the following phenotypes: Biofilm production (**a**), Sliding motility (**b**) Colony morphology (**c**). The same strains were streaked on LB agar plates supplemented with or without Rifampicin (10 μg/ml) to evaluate the Antimicrobial susceptibility towards this antibiotic (**d**)

The *msmeg_0412* gene (Fig. 1a) belongs to a gene cluster devoted to the biosynthesis and transport of GPLs to mycobacterial cell wall surface. Most of the genes inside the cluster have been previously characterized [5, 15]. In particular, *mps1* and *mps2* genes encode the mycobacterial peptide synthetase required for the formation of tripeptide-amino-alcohol moiety which is then linked to the 3-hydroxy/methoxy C26-C34 acyl chain, the lipid moiety of GPLs. The latter is synthesized by the combined action of Pks, Fad23 and PapA3 gene products. Finally, the action of various glycosyltransferases (Gtf), methyltransferases (Mtf) and acetyltransferases (Atf) leads to the building of the glycosyl side of GPLs. Once synthesized, GPLs are transferred to GPL addressing protein (Gap), a small integral membrane protein, required for the translocation of GPLs on the cell surface. As shown in Fig. 1a, the *msmeg_0412* gene is close to the genes involved in the synthesis of lipid portion of GPLs. In order to understand the role of the *msmeg_0412* gene product, we constructed a null mutant strain by using the pGOAL/pNIL procedure as reported in methods. A PCR confirmation of gene deletion in GM1 strain is shown in Fig. 1b. The deletion mutant, GM1, was analyzed for some phenotypes . Compared to its isogenic wild type strain, GM1 displayed altered biofilm formation (Fig. 2a), lack of sliding motility (Fig. 2b), and rough colony phenotype (Fig. 2c).

To confirm that these phenotypes were due to *msmeg_0412* gene inactivation, we performed a complementation analysis. A wild type allele of the *msmeg_0412* gene was PCR-amplified, cloned into pMV10-25 vector [27] without the *gfp* gene (methods), and transformed into strain GM1, yielding strain GM2. As shown in Fig. 2 all phenotypes of strain GM1 were rescued in strain GM2, that appeared identical to the wild type strain.

The drastic impact of *msmeg_0412* deletion on *M. smegmatis* surface properties was also evidenced by a slower growth rate in liquid media with a generation time of the mutant of 4.5 h compared to 3 h of the wt strain (see Additional file 1: Figure S1) and by the enhanced susceptibility to antimicrobials. As shown in Fig. 2d, when streaked on LB agar medium containing Rifampicin at a sub-MIC concentration (10 μg/ml) [28], the GM1 strain was unable to grow compared to the wild type and complemented strains. The same result was obtained using Erythromycin as antibiotic (13 μg/ml) [29]. Since both antibiotics have cytosolic targets, it is possible to hypothesize that the mutation induces changes in the cell wall permeability that lead to an increase in antibiotics penetration.

**Fig. 3:**
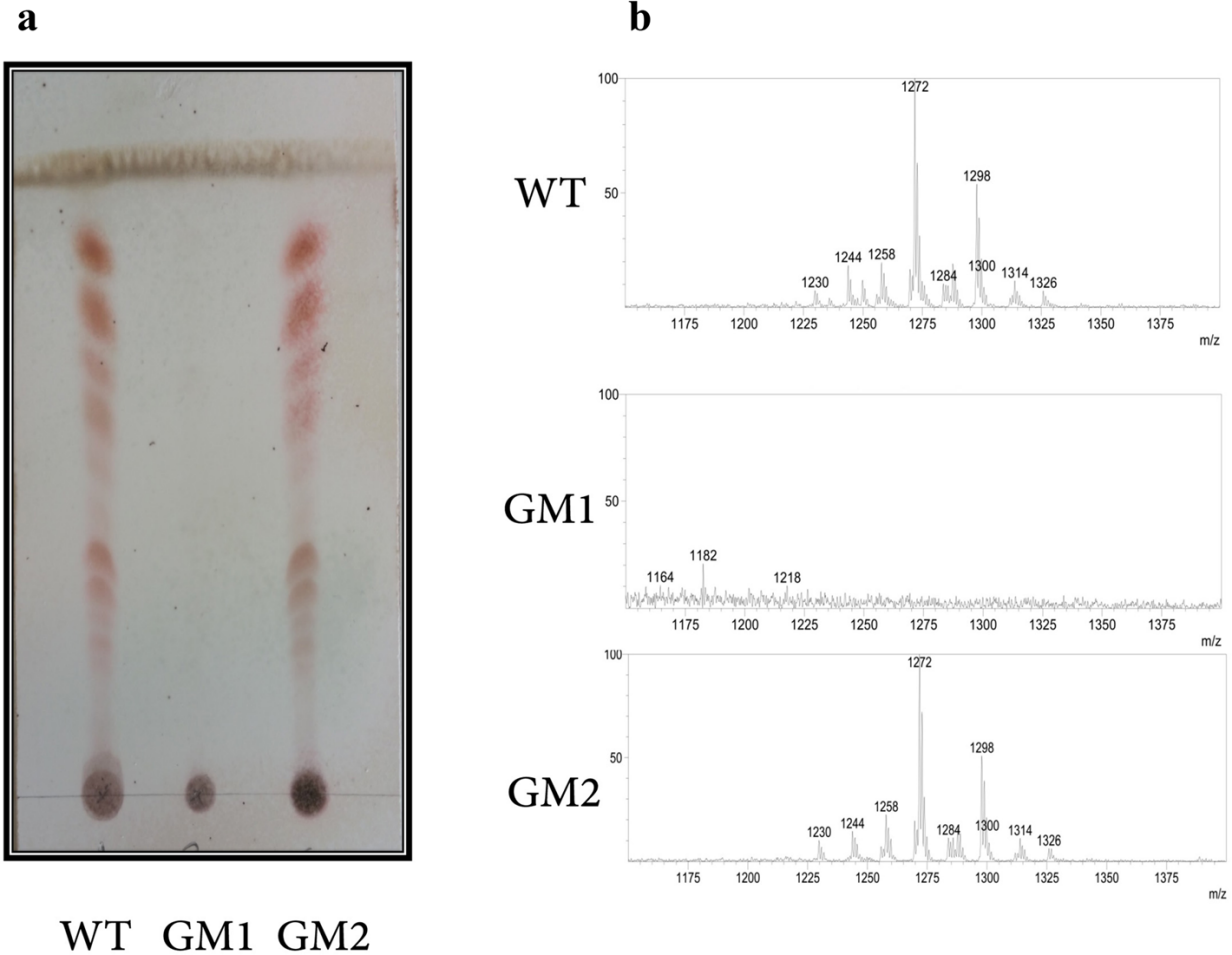
Chemical analysis of GPLs. GPLs content from the wild-type, GM1 and GM2 strains as indicated by TLC analysis (**a**) and ESI-MS profile of the organic extracts (**b**)

To better characterize the effects of *msmeg_0412* deletion we extracted and analyzed the GPLs content from the wild type, mutant and complemented strains. TLC analysis of the three organic extracts (Fig. 3a) indicated that GPLs were absent only in GM1 while they were equally abundant in the remaining strains. Conversely, the occurrence of the lipid fraction was detected in both wt, GM1 and GM2 strains (see Additional file 2: Figure S2). An even more accurate picture was provided by ESI-MS analysis (Fig. 3b). In this case, wt and GM2 strains provided identical MS profiles, which could be referred to the occurrence of 6-deoxy-l-talose- and *O*-methyl-l-rhamnose-containing GPLs and their methylated congeners [23]. Conversely, no traces of any sugar-containing compound at the same *m/z* range was found in GM1. The lack of GPLs in the *msmeg_0412* deletion mutant apparently suggests that *msmeg_0412* gene product is involved in the synthesis and assembly of GPLs on the *M. smegmatis* cell wall.

### The *msmeg_0412* gene expression is induced by lipid substrates

**Fig. 4:**
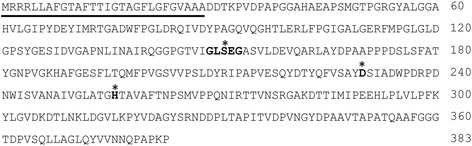
Amino acid sequence of MSMEG_0412. In bold is reported the conserved pentapeptide sequence motif GXSXG/S. Aminoacid residues potentially involved in the catalytic triad are indicated by an asterisk (*). Underlined is the putative N-terminal signal peptide

In order to evaluate the role played by MSMEG_0412 protein during GPLs production a bioinformatics approach was initially followed. MSMEG_0412 has the canonical penta-peptide sequence motif GXSXG/S and the conserved Ser, Asp and His catalytic triad (indicated in bold in Fig. 4) typical of proteins with lipase/esterase activity and identified as hydrolases involved in lipid metabolism [30, 31].

**Fig. 5:**
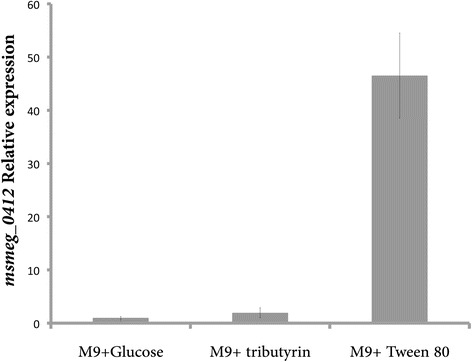
Real-Time PCR analysis of *msmeg_0412* gene under different growth conditions. Cells of *M. smegmatis* mc^2^155 wild type strain were grown in aerated conditions in minimal medium containing glucose, tributyrin or tween 80 to early exponential growth phase. The ∆∆Ct method was used to calculate the relative amount of specific RNA present in each sample, and the transcriptional induction determined by comparison to values from cells growing in minimal medium containing glucose

To experimentally validate the bioinformatic prediction we monitored the effects of two widely used lipid substrates, tributyrin and tween 80, on the transcriptional profile of the *msmeg_0412* gene. To this purpose *M. smegmatis* wild type strain was grown in minimal medium containing glucose, tributyrin or tween 80 as the only carbon source and total RNA was extracted and reverse transcribed as reported in methods. As shown in Fig. 5, the transcriptional level of the gene was induced approximately 50-fold in the presence of tween 80 and 2-fold in the presence of tributyrin.

Based on this result we decided to evaluate the effect of tween 80 on the growth of the mutant strain. To this purpose wt, GM1 and GM2 strains were grown in minimal medium containing 0.2 % (w/v) glucose or 1%t ween 80 as the only carbon source. The result (see Additional file 3: Figure S3) clearly indicated that the mutant was unable to grow in presence of tween 80. Although this result, together with the bioinformatic analysis, allows speculating a lipolytic function for MSMEG_0412, a detailed biochemical characterization is needed to decipher its function.

### The MSMEG_0412 protein is localized on the cell wall

**Fig. 6:**
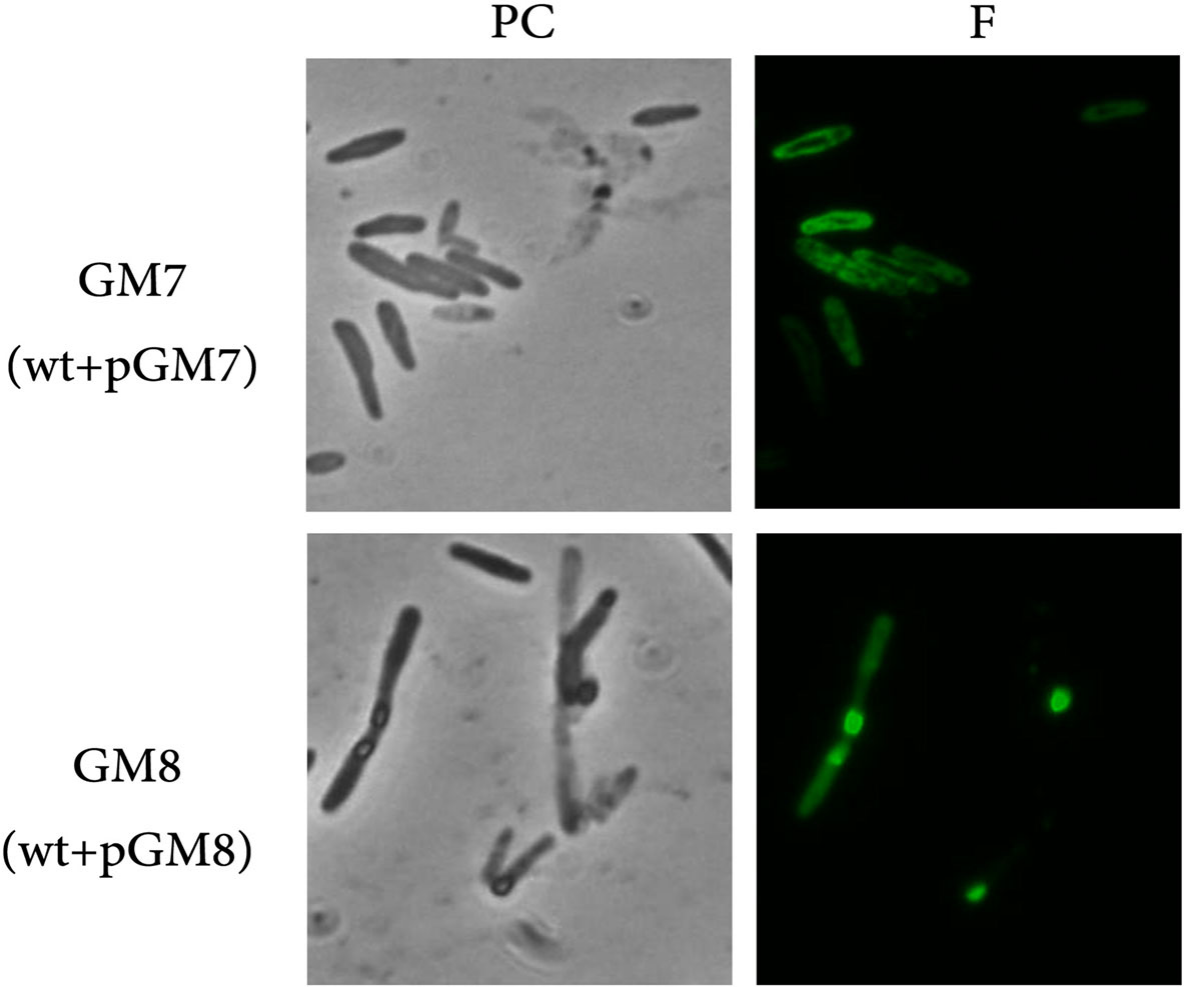
Fluorescence analysis. *M. smegmatis* strains carrying gene fusions between *gfp* and *msmeg_0412* gene with (GM7) and without (GM8) the region coding for the putative signal peptide were analysed by phase contrast (PC) and Fluorescent (F) microscopy. Exposure time was 200 ms in both cases

*In silico* analysis suggested that MSMEG_0412 contains a N-terminal signal sequence (underlined in Fig. 4) with a putative cleavage site between residues A29 and D30, most likely involved in targeting the MSMEG_0412 to the cell membrane. To validate this prediction we constructed two gene fusions between *gfp*, the gene encoding for the Green Fluorescent Protein, and the *msmeg_0412* gene with and without the sequence coding for the putative signal peptide (Methods). The gene fusions were independently used to transform *M. smegmatis* wild type cells, yielding strains GM7 (carrying the entire *msmeg_0412* gene) and GM8 (carrying the *msmeg_0412* gene without the signal peptide). A fluorescence microscopy analysis showed that the fluorescence signal was localized around the cells of strain GM7 and inside the cells of strain GM8 (Fig. 6). This result indicates that MSMEG_0412 is a membrane protein and that the removal of the signal peptide induces protein aggregation and its retention within the cytosol.

## Discussion

Mycobacteria comprise bacilli with a specialized cell wall divided in two compartments: the inner- and the outer- layer. The latter is rich in lipids and glycolipids which differ among species. *M. smegmatis* shares a richness in glycopeptidolipids (GPLs) with pathogenic non tubercle mycobacteria, such as *M. abscessus and M. avium.*

*Msmeg_0412*, a gene located in the GPLs biosynthetic locus, is highly conserved among mycobacteria, and its product shares 45 % identity with Rv1184c from the human pathogen *M. tuberculosis.* The gene encodes a protein which primary structure has features of proteins annotated as putative lipases or esterases.

Lipase/esterase-like enzymes belong to a family of proteins involved in lipid metabolism, which play a crucial role during mycobacterial infection and persistence inside the host [30, 32]. In fact, it has been reported that such enzymes contribute to the destruction of the host tissues thereby supplying nutrients to the pathogens [33, 34]. Therefore, a better understanding of these proteins will surely help in developing new strategies against mycobacterial infections.

Earlier reports have clearly indicated that the loss of GPLs on *M. smegmatis* surface induce an increased cell wall hydrophobicity [14, 15, 23] which results in the alteration of surface properties such as a change of colony morphotype (from smooth to rough), an altered biofilm formation at liquid/air interface, and the inability to slide on soft agar plates. Here we report the construction and analysis of a *M. smegmatis* strain carrying a null mutation in *msmeg_0412* gene.

Our results show that MSMEG_0412 is required for the correct processing of the GPLs on mycobacterial surface and that its deletion induced a surface perturbation. Furthermore, in line with the bioinformatic predictions, the *msmeg_0412* gene expression is enhanced in the presence of lipid substrates, and the protein encoded localizes on the cell wall. Taken together our results allow to speculate that once synthesized, MSMEG_0412 is translocated to the cell wall, where plays a crucial role for the correct GPLs processing and translocation on *M. smegmatis* surface. A further biochemical approach is needed to better clarify other aspects of the function and the localization of this important protein.

## Conclusions

The analysis of *msmeg_0412* mutant highlighted that this gene is important for *Mycobacterium smegmatis* surface organization and resulted in different important phenotypes, as lack of sliding motility, altered biofilm formation and enhanced drug susceptibility. The analysis of Glycopeptidolipids (GPLs) component of bacterial surface showed that the *msmeg_0412* mutant was strongly impaired for GPLs presence in the mycobacterial cell wall. In addition, based on *in-silico* predictions, we report that the expression of the gene is enhanced by the presence of lipidic substrates and that the encoded protein has a membrane localization.

Finally, generation time of the mutant was shorter than the one of the wild type strain.

Mutations affecting the organization of the cell wall have often pleiotropic effects. This is the first experimental evidence for an involvement of *msmeg_0412* gene in the synthesis of the GPLs.

Taken together, the results presented in this manuscript offer the possibility to consider MSMEG_0412 as a new target to impair the mycobacterial growth and to increase drug susceptibility.

## Methods

### Bacterial strains, media and growth conditions

*M. smegmatis* mc^2^155 [35] was the parental strain of all the recombinant strains described below. DH5α (*supE44 ΔlacU169 [ϕ80ΔlacZM15] hsdR17 recA1*) [36] was used for all cloning experiments.

The recombinant strain GM1, carrying a *msmeg_0412* gene deletion was engineered using the p2NIL/pGOAL19-based flexible cassette method as previously reported [37].

*M. smegmatis* mc^2^155 and derivatives were grown in 7H9 medium (Difco) containing 2 % glycerol and 0.05 % tween 80 (Applichem) or in M9 containing 1 mM Mg_2_SO_4_ and 0.2 % glucose. Where indicated, glucose was replaced by 1 % glycerol-tributyrate (tributyrin) or 1 % tween 80.

*E. coli* strains were grown in LB medium. When required, antibiotics were added to the medium at the following final concentrations: ampicillin 100 μg/ml, hygromycin at 200 μg/ml for *E. coli* and 50 μg/ml for *M. smegmatis* mc^2^155, respectively.

For the antimicrobial susceptibility test, Erythromycin and Rifampicin were added to LB agar medium at the following final concentrations: 13 μg/ml and 10 μg/ml respectively.

For the Microscopy analysis, samples were observed using an Olympus BX51 fluorescence microscope using a FITC filter. The Images were captured using an Olympus DP70 digital camera and processed. Standard acquisition times were 200 ms.

#### Bioinformatic approach

A multiple sequence alignment was carried out using PRALINE (http://www.ibi.vu.nl/programs/pralinewww) as program. MSMEG_0412 aminoacid sequence was used as query against the earlier identified *M. tuberculosis* proteins [31] showing the conserved pentapetide sequence motif (GXGXG) and the conserved Ser, Asp and His catalytic triad characteristic of lipase/esterase activities.

The signal peptide server, (http://www.cbs.dtu.dk/services/SignalP), was used to predict the presence and location of the signal peptide cleavage site inside MSMEG_0412.

### Sliding motility assay

5 μl of a liquid culture in stationary phase was dispensed on plate containing 7H9 medium plus 0.3 % agar with no added carbon source. The plate was then incubated at 37 °C for 1 week [15, 23].

### Congo red assays

5 μl of a liquid culture in stationary phase was dispensed on plate containing LB medium supplemented with 2 % agar and 100 μg/ml Congo Red (Sigma) and incubated at 37 °C for 3 days [15, 23].

### Biofilm assay

5 μl of a liquid culture in stationary phase was inoculated into PVC well plates containing 5 ml Sauton liquid medium and incubated in standing condition at 37 °C for 1 week [15, 23].

### DNA manipulation

Plasmid and chromosomal DNA preparation, restriction digestion, ligation, bacterial transformation and agarose gel electrophoresis were performed as described [36].

Plasmid pGM5 carries a gene deletion cassette (Δ0412 cassette) containing 689-bp segment upstream of *msmeg_0412* gene + first 4 codon (5’ fragment) followed by last 15 codons + 685-bp segment downstream of the gene (3’ fragment). The 5’ fragment was PCR amplified using the primer pairs 0412-1 F and 0412-1R listed in Table 1 and the genomic DNA of wild type mc^2^155 strain as template and the obtained PCR product was cloned in pGEMT-easy vector, giving the plasmid pGM2. The 3’ fragment was PCR amplified using the primer pairs 0412-2 F and 0412-2R listed in Table 1 and the genomic DNA of wild type mc^2^155 strain as template and the obtained PCR product was digested with *KpnI* and cloned in pGM2 vector at the unique *KpnI* restriction site, giving plasmid pGM3. The deletion cassette was then excised from pGM3 vector using the restriction enzymes *BamHI* and *SalI* and transferred into the p2NIL plasmid previously digested by the same restriction enzymes. The resulting plasmid, named pGM4 and the plasmid pGOAL19 were digested with *PacI*, and the *PacI* selectable marker cassette of pGOAL19, carrying the *sacB*, *LacZ* and *Hyg* genes, was ligated to the linearized pGM4 to create the suicide vector pGM5.

**Table 1:** Synthetic oligonucleotides

Name	Sequence (5′ - 3′)^a^	Position of Annealing^b^
0412-S	CATGATCCCCGAGGAGCAC	+857/+875
0412-A	TTGCCGTTCAAGTACCTCGG	+889/+90
0412-Fw	AAAGCTAGCATGAGGAGACGACTTCTCGCGTTC	+1/+24
0412-spFw	AAAGCTAGCGATGACACCAAACCGGTCG	+85/+103
0412-Rv	AAAGCTAGCTCAGGGCTTGGGCGCCGGTTG	+1132/+1152
0412-gfpRv	AAAGCTAGCGGGCTTGGGCGCCGGTTG	+1132/+1149
0412-1F	CTAGAAGCTTGCACCGCACTGGTGAGATA	-690/-672
0412-1R	CTAGGGTACCCGCTGTAGTCCTCTGCT	-5/+12
0412-2F	CTAGGGTACCGGACTGCAGTACGTCGTGAACA	+1108/+1129
0412-2R	CTAGGGTACCACCATGTGGATCCCTCTCCT	+1818/+1837

^a^Underlined is an unpaired tail carrying a restriction site

^b^Position of annealing refers to *msmeg_0412* gene sequence, with the first base of the translational initiation codon as +1

Plasmid pGM6, used for complementation analysis, was constructed as follow: the wild type *msmeg_0412* gene from *M. smegmatis* wild type strain was PCR amplified with *Pfu* Turbo high Fidelity DNA polymerase (Stratagene) using chromosomal DNA as template and the oligo 0412-Fw and 0412-Rv (Table 1) as primers. The last ones contained the engineered *NheI* and *BamHI* restriction sites respectively.

The PCR product of expected size was purified with a PCR Purification Kit (Qiagen), digested with *NheI* and *BamHI* restriction enzymes and cloned under the *hsp60* promoter into the shuttle vector pMV10-25 [27], previously digested with the same enzyme pairs which allowed the excision of the *gfp* encoding gene. The resulting plasmid was controlled by sequencing and then used to transform GM1 cells, giving the strain GM2.

Plasmid pGM7 and pGM8 containing the entire version or the truncated version of the *msmeg_0412* gene fused to the *gfp* reporter gene, respectively, were constructed as follow: the *msmeg*_*0412* gene except the stop codon was PCR amplified with *Pfu* Turbo high Fidelity DNA polymerase (Stratagene) using chromosomal DNA as template and priming the reaction with two different couples of primers: 0412-Fw and 0412-gfpRv or 0412-spFw and 0412-gfpRv (Table 1). The first couple of primers has allowed to amplify the entire copy of the gene, the other combination of primers has allowed to amplify the gene that misses of 84 bp, encoding a putative N-terminal signal peptide. All primers contained a 5’ engineered *NheI* restriction site. The PCR products of expected sizes were purified with the PCR purification Kit (Qiagen) digested with the *NheI* restriction enzyme and cloned in the plasmid pMV10-25 [27], under the control of *hsp60* promoter and *in frame* with GFP coding gene using the *NheI* restriction site located at 5’ end of *gfp.*

The resulting plasmids were controlled by sequencing and then used to transform *M. smegmatis* wild type cell, giving the strains GM7 and GM8 respectively.

### Isolation of Δmsmeg_0412 mutant strain

The *msmeg_0412* null mutant strain, named GM1, was obtained by using the allelic replacement system reported previously [37].

The pGM5 suicide vector was used to transform cells of *M. smegmatis* mc2155 strain and transformants were selected on LB agar plates supplemented with hygromicin (200 mg/ml), kanamycin (25 mg/ml) and X-Gal (50 mg/ml). Blue colonies were restreaked onto LB agar plates; a loopful of cells was then resuspended in LB broth, serially diluted and plated on LB agar containing 2 % sucrose (Suc) and X-gal. White Kan^S^, Hyg^S^ and Suc^R^ colonies were isolated and identified by PCR analysis. One Kan^S^, Hyg^S^ and Suc^R^ colony with a deleted *msmeg_0412* gene was identified as GM1 strain.

### RNA extraction

Total RNA was extracted by using the RNeasy Mini Kit (QIAGEN) according to manufacturer’s protocol. RNAs were resuspended in 100 ml of RNAse-free water and treated with DNAse I (Fermentas, 1U/ml) for 30 minutes at 37 °C. The quality of RNA samples were estimated using the RNA nanochip on the Thermoscientific Nanodrop 1000. The concentration of RNA was determined by measuring the absorbance at 260 nm.

### Real Time PCR analysis

cDNA was synthesized by using PrimeScript Enzyme mix (Takara) and random hexamers as primers. Primers 0412-S and 0412-A (Table 1), and those for the normalizing gene 16S, 16S-fw (5′-GGCGAACGGGTGAGTAACA-3′) and 16S-Rv (5′-GCCCTGCACTTTGGGATAAG-3′), were designed with ABI PRISM Primer Express software (PE Applied Biosystems). Real-Time PCR was performed by using SYBR green RT-PCR (Takara). Reactions were performed as previously described [38]. Values reported here were the average of at least three independent experiments and were expressed as the mean ± SEM (Standard Error of the Mean). The fluorescence signal due to SYBR Green intercalation was monitored to quantify the double-stranded DNA product formed in each PCR cycle. Statistical significance was determined by Student’s unpaired *t*-test and the significance levels were reported in the text.

### Extraction and analysis of mycobacterial lipids

Total lipids were extracted from whole cells as previously reported [16]. In brief, cells from wt, mutant and complemented strains were grown in LB medium until stationary phase (OD_600_: 2.5), harvested 10 minutes at 7000 rpm and resuspended in PBS, pH 7.4. Lipids were extracted with CHCl_3_:CH_3_OH (1:2 v/v) for 24 hours, separately dried under vacuum and partitioned between water and chloroform (1:1 v/v).

The organic phases were further washed with distilled water and evaporated to dryness. For analytical thin-layer chromatography (TLC), the crude residues (1.0 mg) were dissolved in CHCl_3_:CH_3_OH (0.75 mL, 9:1 v/v), spotted on silica gel 60-precoated plates (0.25-mm thickness, Merck) and eluted with CHCl_3_:CH_3_OH (9:1 v/v). Sugar-containing compounds were visualized by spraying TLC plates with a chromosulfuric acid staining solution and charring them at 200 °C for 2 min. The GPLs were detected as brownish-red spots. Conversely, the lipid content in the three strains was detected by extracting the crudes with hexane (0.3 mL). The resulting solutions were spotted on TLC plates which were eluted in toluene:acetone (99:1 v/v). In this case, lipids were visualized by spraying the plates with a cerium molybdate staining solution and charring them at 200 °C for 2 min. The lipids were detected as dark blue spots.

ESI-MS analysis of the organic extracts was performed using a Shimadzu LC-MS-2010EV system with ESI interface, Q-array-octapole-quadrupole mass analyser and a Shimadzu LC-MS solution Workstation software for data processing. The optimized MS parameters were selected as follows: CDL (curved desolvation line) temperature 250 °C; heat block temperature 250 °C; probe temperature 250 °C; detector gain 1.6 kV; probe voltage +4.5 kV; CDL voltage -15 V. Nitrogen served as nebulizing (flow rate: 1.5 L/min) and drying gas (flow rate: 15 L/min). Each sample (0.5 mg) was dissolved in 1 mL of acetonitrile (ACN)/0.1 % trifluoroacetic acid (TFA); 2 μL aliquots of the resulting solutions were injected using ACN/0.1 % TFA as solvent (flow rate: 0.2 mL/min). MS spectra were acquired in positive ion mode.

## Additional files

Additional file 1: Figure S1. Growth curve of *M. smegmatis* strains in LB medium. *M. smegmatis* wild type and GM1 strains were grown in LB medium containg 0,05 % tween 80 and OD_600nm_ determined every 3 hours. For each strains the data reported in graph are the mean of three independent experiments. (JPG 601 kb)
Additional file 2: Figure S2. Chemical analysis of total lipids. Total lipids were extracted from whole wt, GM1 and GM2 cells and analyzed by TLC. (JPG 483 kb)
Additional file 3: Figure S3. Growth curve of *M. smegmatis* strains in Minimal medium. *M. smegmatis* wild type, GM1, GM2 strains were grown in minimal medium containing 0.2 % (w/v) glucose or 1%t ween 80 as the only carbon source. and OD_600nm_ determined every 3 hours. For each strains the data reported in graph are the mean of three independent experiments. (JPG 710 kb)

## Abbreviations

ESI-MS: Electrospray Ionization Mass Spectometer
GPLs: Glycopeptidolipids
TLC: Thin Layer chromatography
