# Supplementary figures and images for: Inactivation of MSMEG_0412 gene drastically affects surface related properties of Mycobacterium smegmatis
Source: BMC Microbiol. 2016 Nov 8;16:267. doi: 10.1186/s12866-016-0888-z (PMC5101647; doi:10.1186/s12866-016-0888-z)

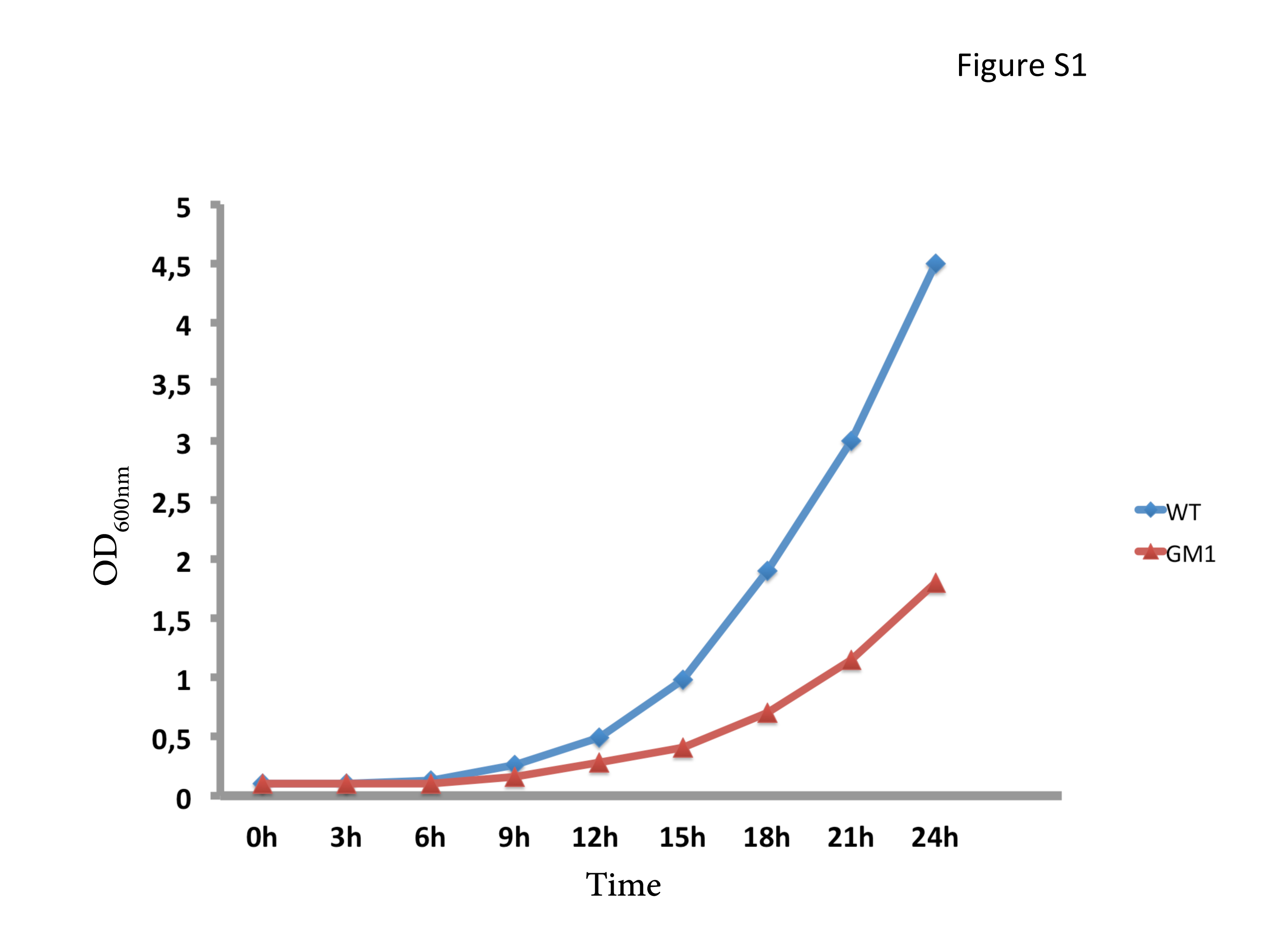

Supplement: Additional file 1: Figure S1. — Growth curve of M. smegmatis strains in LB medium. M. smegmatis wild type and GM1 strains were grown in LB medium containg 0,05 % tween 80 and OD600nm determined every 3 hours. For each strains the data reported in graph are the mean of three independent experiments. (JPG 601 kb) [file 12866_2016_888_MOESM1_ESM.jpg]

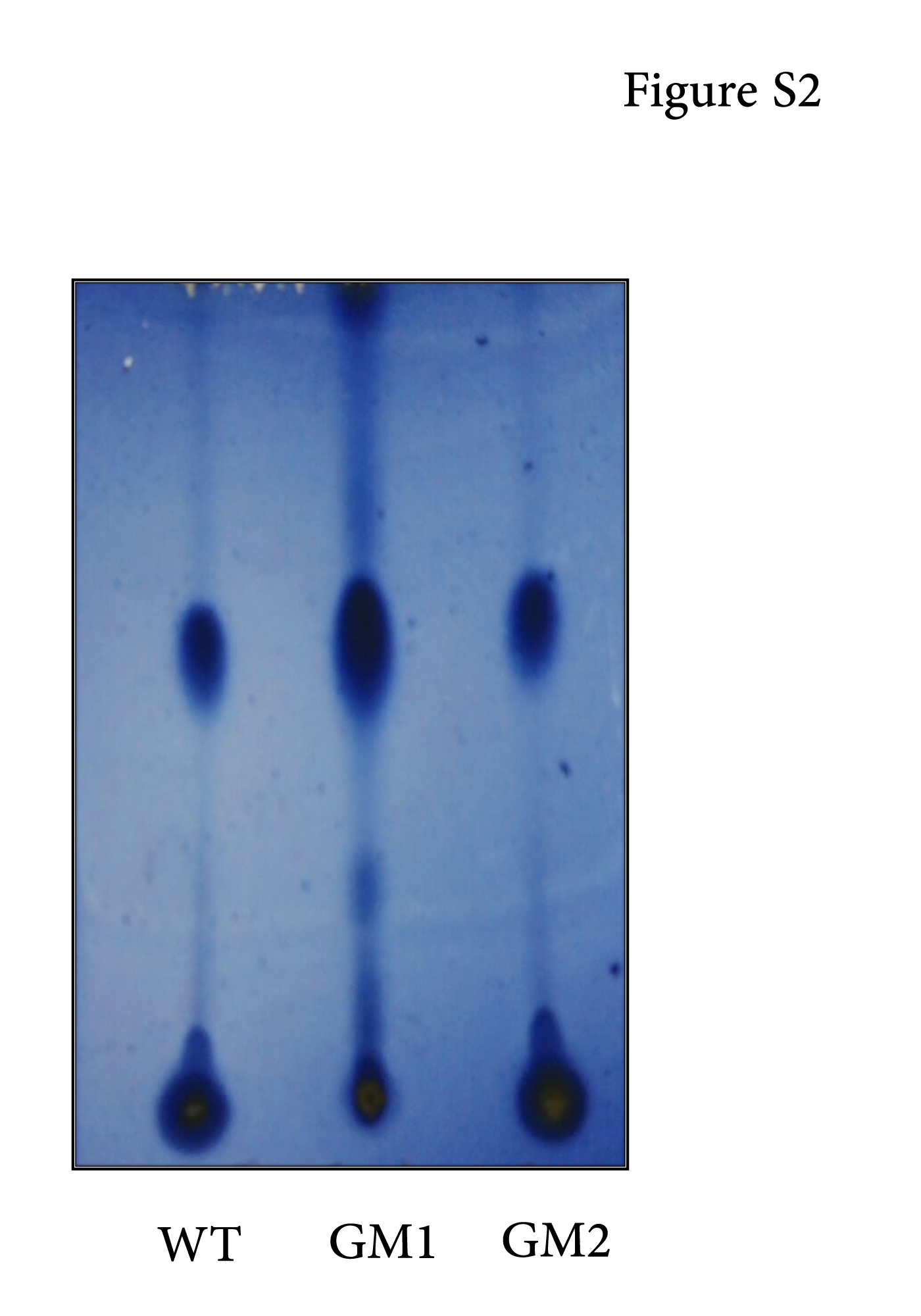

Supplement: Additional file 2: Figure S2. — Chemical analysis of total lipids. Total lipids were extracted from whole wt, GM1 and GM2 cells and analyzed by TLC. (JPG 483 kb) [file 12866_2016_888_MOESM2_ESM.jpg]

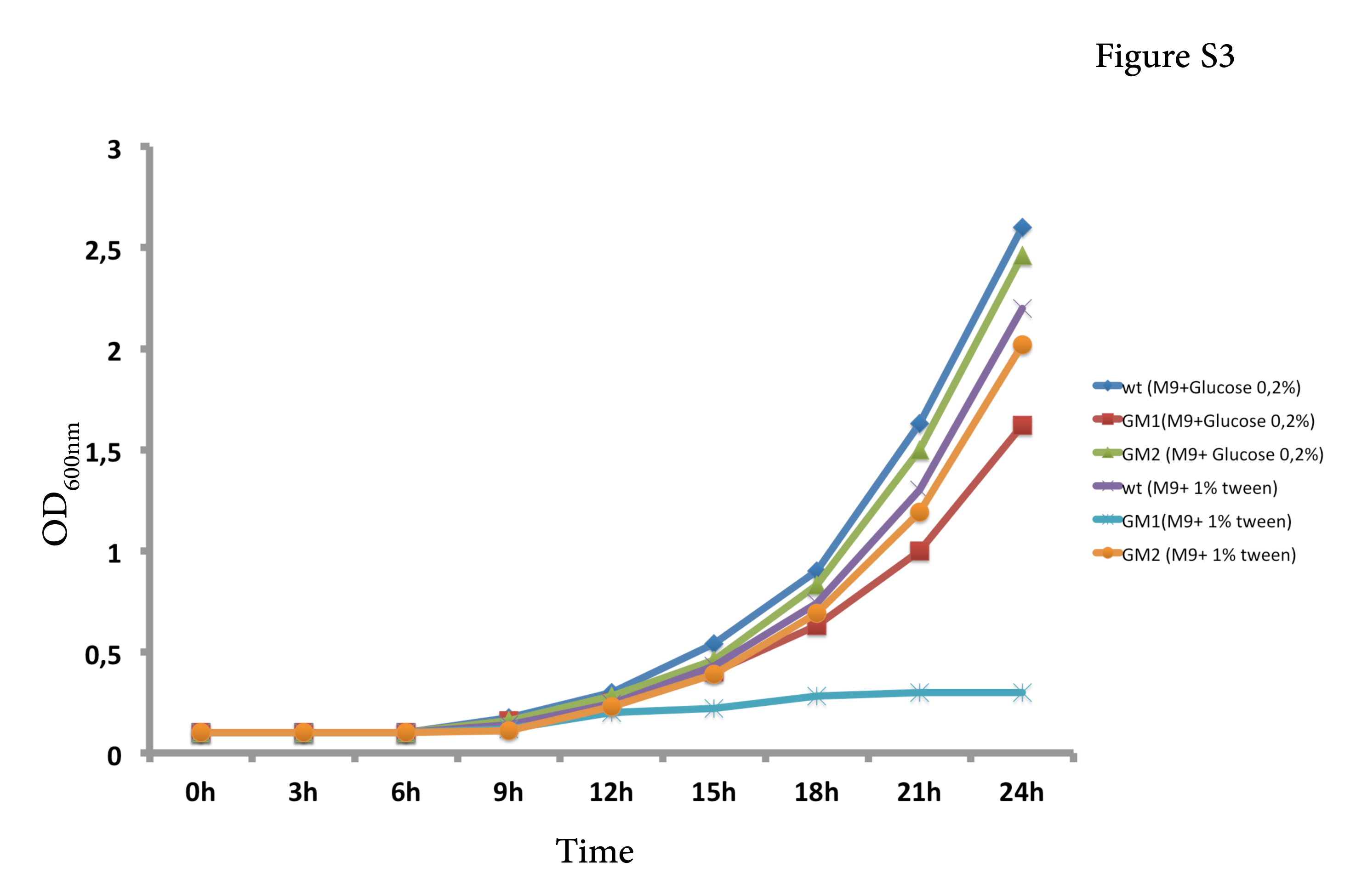

Supplement: Additional file 3: Figure S3. — Growth curve of M. smegmatis strains in Minimal medium. M. smegmatis wild type, GM1, GM2 strains were grown in minimal medium containing 0.2 % (w/v) glucose or 1%t ween 80 as the only carbon source. and OD600nm determined every 3 hours. For each strains the data reported in graph are the mean of three independent experiments. (JPG 710 kb) [file 12866_2016_888_MOESM3_ESM.jpg]
